# Influence of Fuel–Air Ratio, Exhaust Temperature, and Atmospheric Absorption Effects on Infrared Spectral Features of Aero-Engine Wake

**DOI:** 10.3390/s26196167

**Published:** 2026-09-29

**Authors:** Haonan Li, Yurong Liao, Chen Cheng, Zhenping Kang, Shuping Huang, Rui Feng

**Affiliations:** 1 Department of Electronic and Optical Engineering, Space Engineering University, Beijing 101416, China; lhn_links@hgd.edu.cn (H.L.);; 2 Anhui Province Key Laboratory of Optical Quantitative Remote Sensing, Hefei Institutes of Physical Science, Chinese Academy of Sciences, Hefei 230031, China

**Keywords:** aero-engine wake, infrared spectral feature, fuel–air ratio, exhaust temperature, atmospheric absorption effect

## Abstract

The infrared spectral features of aero-engine wake serve as a crucial basis for infrared detection, tracking, and identification of aircraft. Their spectral line structure is regulated by the concentration and temperature of the exhaust gases, as well as atmospheric absorption effects. This study employs a high-precision numerical simulation model and conducts field experiments on infrared spectral detection of exhaust plumes to systematically investigate the influence mechanisms of three factors—fuel–air ratio (achieved by varying exhaust gas concentration), exhaust temperature, and atmospheric absorption effects—on key spectral features, including peak intensity, line broadening, shape, and position. Results indicate the following. The fuel–air ratio enhances peak intensity and broadens spectral line width by increasing CO_2_ and H_2_O concentrations within the fuel-lean operating regime (f = 0.025–0.031); exhaust temperature causes exponential spectral intensity growth and line broadening by elevating molecular energy level populations, while inducing H_2_O spectral band redshifts and fine-structure evolution; selective absorption by atmospheric H_2_O, CO_2_, and O_3_ causes severe spectral attenuation near bands at 1041 cm^−1^, 1590 cm^−1^, 2349 cm^−1^, and 3756 cm^−1^, with transmittance distribution modulated by geographical and seasonal factors. This study provides theoretical support for wake spectral feature extraction, infrared stealth design, and detection/tracking identification.

## 1. Introduction

The aero-engine wake, due to its distinct infrared spectral features that differ markedly from other aircraft components and the surrounding environment, has become a key “fingerprint” for infrared detection, tracking, and identification of aircraft [1,2,3]. Its spectral features—including line peak intensity, broadening, shape, and position—are directly determined by the internal combustion chemical reactions within the plume and the external atmospheric absorption effects. In this process, the fuel–air ratio, as the core input parameter of the combustion system, directly determines combustion completeness and the concentration of characteristic gas components. The exhaust temperature, serving as the initial thermodynamic condition, significantly influences fluid properties and molecular transition probabilities. Furthermore, the selective absorption of atmospheric gases such as H_2_O, CO_2_, and O_3_ further alters the spectral structure and intensity distribution of the wake radiation [4,5,6]. These three factors result in complex band selectivity and parameter sensitivity in the infrared spectrum of the exhaust plume, necessitating systematic single-factor analysis to reveal their individual influence mechanisms.

Early pioneering studies on exhaust plume infrared radiation date back to the 1980s. Decher [7] numerically investigated the infrared emission from turbofan engines with high aspect ratio nozzles, revealing the influence of nozzle geometry on the plume infrared signature. Nelson [8] analyzed the influence of particulates on the infrared emission from tactical rocket exhausts. Heland and Schäfer [9] first applied Fourier-transform infrared (FTIR) emission spectroscopy to the analysis of aircraft exhausts, and subsequently determined the major combustion products in aircraft exhausts by FTIR emission spectroscopy [10]. Surzhikov [11] developed a three-dimensional model of the spectral emissivity of light-scattering exhaust plumes. Buzykin et al. [12] reported the spectroscopic detection of sulfur oxides in the aircraft wake, and Dix et al. [13] conducted an infrared signature reduction study on a small-scale jet engine. These early works laid the experimental and modeling foundations for the wake infrared spectral analysis presented in this study.

Regarding the influence of component concentration, Wang et al. [14] conducted ground static tests comparing the radiation intensity of wakes from dual-base propellants with different energy characteristics. They demonstrated that fuel composition and fuel–air specific energy alter characteristic gas concentrations, thereby regulating radiation signals in the 2.2–10 μm band. Niu et al. [15] developed an IRSAT model to investigate the effect of HTPB/AP ratio on rocket exhaust infrared radiation, finding that a 3% increase in HTPB mass fraction could elevate radiation intensity by up to 50% in certain bands. They attributed this variation primarily to changes in exhaust gas concentrations of H_2_O, CO_2_, and other components. Cai et al. [16] demonstrated that elevated concentrations of radiative components like H_2_O and CO_2_ in the exhaust plume during reignition reactions cause radiation intensity in the 2–5 μm band to increase by 50–100% under single-step reactions and 150–170% under multi-step reactions; Bao et al. [17] validated through a 17-step chemical reaction mechanism model combined with ground test data that elevated rocket engine exit temperatures significantly enhance flame re-ignition effects. This increases the concentration of radiatively active components like H_2_O and CO_2_ in the wake, leading to substantial increases in infrared radiation intensity at wavelengths such as 2.7 μm and 4.3 μm. Zhou et al. [18] simulated infrared radiation from typical aero-engine exhaust systems using a multi-scale narrow-band-correlated k-distribution model. They found that CO_2_ contributes significantly more to wake infrared radiation intensity than water vapor in both the 3–5 μm and 8–14 μm bands, and that lower carbon content in fuel correlates with reduced wake infrared radiation intensity.

Regarding temperature effects, Sun et al. [19] analyzed exhaust temperature uncertainty using polynomial chaotic expansion, concluding that elevated temperatures increase the probability of radiation transitions in H_2_O characteristic bands and cause peak redshifts. Jiang et al. [20] investigated changes in CO_2_ molecular vibrational–rotational energy levels at different temperatures, discovering that elevated temperatures alter the peak intensity and fine structure of CO_2_ radiation in the 4.3 μm characteristic band, thereby influencing the infrared radiation characteristics of the wake in this band. Zhang et al. [21] investigated the combined infrared radiation characteristics of aircraft skin and exhaust plumes, finding that the radiation intensity of the exhaust plume in the 3–5 μm band increased exponentially by 5.1 times as the nozzle inlet temperature rose from 700 K to 1000 K, with the nozzle base exhibiting significantly enhanced backward radiation. Pan et al. [22] observed that when helicopter exhaust temperature increased from 900 K to 1200 K, plume radiation intensity in the 3–5 μm band rose by approximately 100%, while its impact on skin radiation was only 5%. The effect on total infrared radiation was primarily concentrated in the plume. Bonchan et al. [23] experimentally measured the infrared spectral radiation intensity of the wake from a microturbine engine at different measurement positions and with circular and square nozzle configurations. They found that as the distance from the nozzle outlet increased, the mixing of the wake with ambient air caused a temperature decrease, with the largest reduction in radiation intensity occurring in the 2100–2500 cm^−1^ band.

In studies of atmospheric absorption effects, researchers have focused on the attenuation of tail radiation transport by atmospheric gases such as H_2_O, CO_2_, and O_3_. For ground-based detection scenarios, Stowe et al. [24] observed through Fourier-transform infrared spectrometer (FTIR) experiments that H_2_O absorption at 3756 cm^−1^ (O-H stretching vibration) and 1590 cm^−1^ (H_2_O bending vibration) in the atmosphere reduces the spectral peak intensity of ground-detected wakes by 15–20%. For space-based detection scenarios, Zhou et al. [25] numerically simulated atmospheric conditions at different altitudes. They found that as altitude increases, atmospheric density decreases, weakening the absorption effects of H_2_O and CO_2_. Consequently, the effective transmission distance of wake radiation can be extended by 30–50%. Li et al. [3] constructed a simulation model of the infrared characteristics of space-based observation aircraft. Through data validation and analysis of the interaction between wake radiation and atmospheric components in the 2.5–13 μm wavelength band, they found that H_2_O and CO_2_ are the primary components causing atmospheric attenuation. These gases strongly absorb wake radiation at wavelengths such as 2.7 μm and 4.3 μm, leading to significant attenuation and energy loss, which is detrimental to signal acquisition by space-based sensors. Arvind et al. [26] modeled atmospheric infrared characteristics using the LOWTRAN code, concluding that atmospheric absorption attenuation causes most infrared radiation from aircraft wakes to be absorbed. Only radiation in bands such as 4.15–4.20 μm transmits through the atmosphere to reach detectors.

Regarding key factors influencing wake infrared spectral radiation, existing studies predominantly focus on single-parameter analyses. Moreover, investigations into atmospheric absorption effects are largely confined to ground-based scenarios, lacking systematic research on spectral characteristic variations under complex conditions such as space-based detection [27,28]. Additionally, aviation engine testing is costly, and full-scale ground tests and flight tests struggle to cover multi-parameter coupled conditions. Therefore, establishing correlation models between wake infrared spectral features and key parameters such as fuel–air ratio, exhaust temperature, and atmospheric conditions through numerical simulation has become an efficient approach for studying wake characteristics [29,30,31]. This paper aims to systematically investigate, from the perspective of spectral feature extraction, the influence patterns of fuel–air ratio, exhaust temperature, and atmospheric absorption effects on the peak intensity, broadening, shape, and position of infrared radiation spectral lines in the wake. This research not only contributes to refining the theoretical framework of aircraft engine wake radiation but also provides critical technical support for aircraft infrared stealth design and detection system optimization.

This study systematically investigates the individual influence mechanisms of fuel–air ratio, exhaust temperature, and atmospheric absorption effects on the infrared spectral features of aero-engine wake for the first time. The main contents can be summarized as follows:

In summary, this study develops an integrated simulation–experiment framework, combining single-step combustion modeling, k-ε wake flow-field simulation, statistical narrow-band radiation calculation, line-of-sight radiative transfer solution, and FTIR field experiments, to systematically investigate the influence of fuel–air ratio, exhaust temperature, and atmospheric absorption effects on wake infrared spectral features.

## 2. Model

### 2.1. Geometric Model

**Figure 1:**
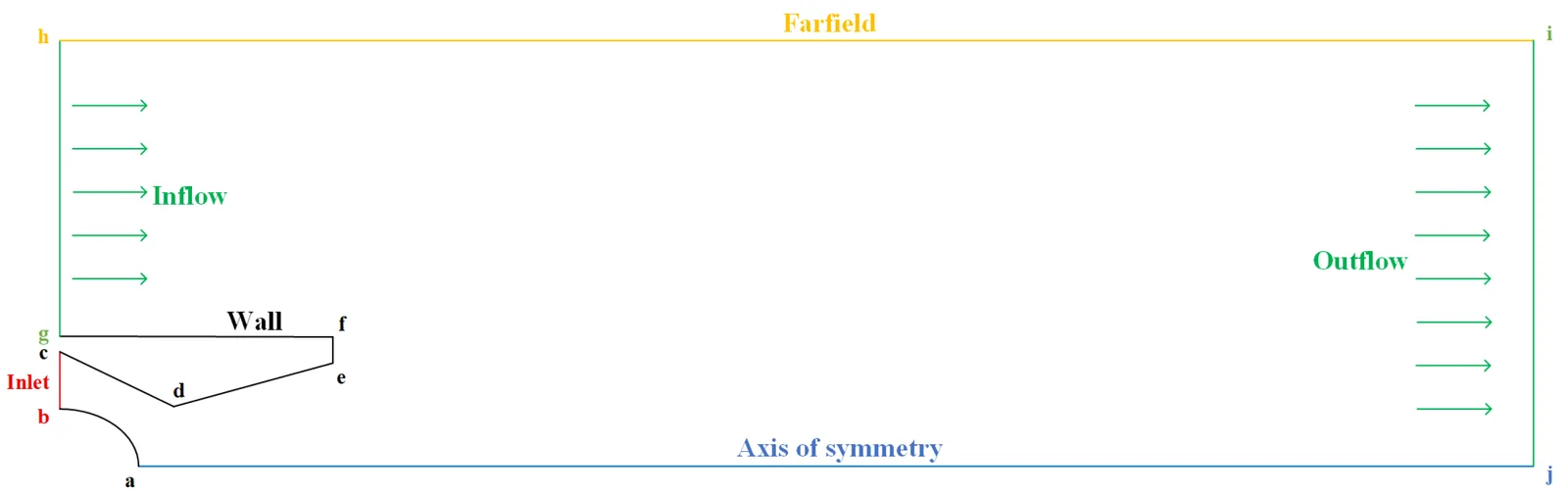
Schematic diagram of boundaries and computational domain.

In the absence of buoyancy, crosswinds, angle of attack, and other external disturbances, the thermal jet from a single engine nozzle can be treated as an axisymmetric structure. The boundary conditions and schematic of the two-dimensional axisymmetric computational domain are shown in Figure 1. The nozzle axis (a–j) is considered an axisymmetric condition. The far-field boundary, inflow boundary, and outflow boundary impose uniform temperature and pressure conditions consistent with free-stream conditions.

The numerical model employs a structured grid. Since the expansion characteristics of the wake vary with temperature and fuel–air ratio, the computational domain and grid must be partitioned based on these parameters. To minimize the impact of grid size on computational results, this study performed mesh independence verification for two sets of conditions: different fuel–air ratios at the same temperature, and different temperatures at the same fuel–air ratio. This resulted in two meshes comprising 200,000 and 220,000 cells, respectively.

### 2.2. Combustion Reaction Kinetic Model

This study primarily focuses on the macroscopic characteristics of the wake’s far field, which exhibits lower sensitivity to nozzle initial conditions compared to detailed chemical reaction pathways. Therefore, to significantly reduce computational complexity, a global single-step reaction model based on equivalence ratios was employed to simulate the combustion of RP-3 aviation kerosene.

C_12_H_23_ was selected as the single-component substitute fuel for RP-3 jet fuel [32]. The combustion products within the engine were modeled and analyzed, with the products of complete combustion being CO_2_ and H_2_O. The specific chemical reaction equations are as follows:

(1) $$C_{12}H_{23}+17.75\left(O_{2}+3.76N_{2}\right)=12CO_{2}+11.5H_{2}O+66.74N_{2}$$

This model assumes complete combustion with no formation of incomplete combustion products such as CO or carbon soot. Thus, the complete combustion of one mole of fuel consumes 17.75 moles of O_2_, producing 12 moles of CO_2_ and 11.5 moles of H_2_O. Nitrogen, as an inert gas, does not participate in combustion. It is evident that the molar quantities of fuel and air directly influence the molar quantities of combustion products. The fuel–air ratio is the core input parameter of this model. Defined as the ratio of fuel mass flow rate to air mass flow rate, it directly determines the completeness of fuel combustion and the distribution of product components in the wake field. Later, we will discuss the influence of this parameter on the gas concentration distribution at the nozzle boundary. For C_12_H_23_/air combustion, the stoichiometric fuel–air ratio is f_st_ = 167.304/(17.75 × 4.76 × 28.97) ≈ 0.068. The fuel–air ratio range investigated in this study (0.025–0.031, i.e., f/f_st_ = 0.37–0.45) lies entirely within the fuel-lean regime, where the assumptions of complete combustion and negligible formation of intermediate species such as CO and soot are physically justified.

### 2.3. Wake Flow Field Model

#### 2.3.1. Fundamental Equations of Fluid Mechanics

This study assumes that the hot jet from an aircraft tail nozzle is a multicomponent, non-uniform ideal gas. The LaminarSMOKE code [33] solves for mass, momentum, component, and energy conservation, assuming the fluid is a Newtonian fluid

(2) $$\frac{\partial \rho }{\partial t}+\nabla \left(\rho v\right)=0$$

(3) $$\frac{\partial }{\partial t}\left(\rho v\right)+\nabla \left(\rho vv+pI\right)=\nabla T+\rho g$$

(4) $$\frac{\partial }{\partial t}\left(\rho Y_{j}\right)+\nabla \left(\rho Y_{j}v\right)=-\nabla \left(\rho Y_{j}V_{j}\right)+{\dot{\Omega }}_{j},\;j=1,\ldots ,N_{C}$$

(5) $$\rho C_{P}\frac{\partial T}{\partial t}+\rho C_{P}v\nabla T=-\nabla q-p\nabla T-\sum_{j=1}^{N_{C}}C_{P,j}Y_{j}V_{j}-\sum_{j=1}^{N_{C}}h_{j}{\dot{\Omega }}_{j}$$

where *t* is time, *ρ* is the mixture density, *p* is pressure, **v** is the mixture velocity, **T** is the fluid stress tensor, **g** is the gravitational acceleration vector, *Y_j_* is the mass fraction of component *j*, **V*_j_*** is the diffusion velocity of component *j*, ${\dot{\Omega }}_{j}$ is the generation rate of component *j*, *T* is the temperature, *C_P_* and *C_P,j_* are the specific heat capacities at constant pressure for the mixture and component *j*, respectively, **q** is the heat flux vector, *h_j_* is the enthalpy of a single component, and *N*_C_ is the total number of species in the kinetic scheme.

The ideal gas equation of state is used to calculate the density of the mixture. The heat flux vector accounts for conduction and radiation

(6) $$q=-\lambda \nabla T+q_{rad}$$

Among these, *λ* is the thermal conductivity of the mixture, and $q_{rad}$ is the radiative heat flux. The diffusion rate is calculated by considering Fickian diffusion and thermal diffusion (the Soret effect)

(7) $$V_{j}=-\frac{D_{j}}{Y_{j}}\nabla Y_{j}-\frac{D_{j}\Theta_{j}}{X_{j}}\frac{1}{T}\nabla T$$

Here, *X_j_* denotes the molar fraction of component *j*, $\Theta_{j}$ represents the thermal diffusivity ratio of component *j*, and *D_j_* is the average diffusion coefficient of the mixture for a single component. This is related to the binary diffusion coefficient *D_j_* and is given by the following expression:

(8) $$D_{j}=\frac{1-Y_{j}}{\Sigma_{i\neq j}^{N_{C}}\frac{X_{j}}{\Gamma_{ij}}}$$

In all simulations analyzed in this paper, the contribution of pressure gradient diffusion to transport is neglected. As proposed by Coffee et al. [34], a forced mass conservation approach is adopted for this topic. This method is based on the so-called mass diffusion velocity $V_{j}^{C}$, which replaces **V***_j_* in Equations (3) and (4) and is defined as follows:

(9) $$V_{j}^{C}=V_{j}+V_{C}$$

where **V_C_** is a constant correction factor independent of components but varying with space and time. This correction factor is introduced to satisfy the law of mass conservation and is calculated as follows:

(10) $$V_{C}=-\sum_{j=1}^{N_{c}}Y_{j}V_{j}$$

#### 2.3.2. Turbulence Model

Turbulence is the core driving force behind the evolution of jet engine wakes, influencing the entire process of wake formation, dispersion, and energy transfer. Crucially, the interaction between the high-temperature wake and the surrounding atmospheric boundary layer determines the scale of the wake and whether secondary combustion effects occur. In the near-field region of the wake, the influence of turbulence fluctuations is negligible due to its supersonic nature; however, in the far-field region, the effects of turbulence become highly significant. Turbulence models exhibit measurement accuracy within 5–10% for radiation, significantly outperforming laminar models with discrepancies reaching up to 20% [35]. Durbin [36] explicitly states that turbulence lies at the core of extensive CFD applications. Consequently, this study employs an implementable two-equation k-ε turbulence model to predict wake features and corresponding flow field distributions.

### 2.4. Statistical Narrow Band Model

The narrow spectral band model relates the arrangement and overlapping properties of spectral lines within a given wavenumber interval Δ*η* (typically 5–50 cm^−1^) to the properties of individual spectral lines. However, it does not require detailed knowledge of the shape, intensity, or position of each line within the spectral band. Instead, it assumes spectral line shapes and posits that their intensity and position distributions follow specific patterns. Depending on the assumed spectral line intensity distribution pattern, statistical spectral band models can be categorized as follows: equal-intensity distribution (random spacing), exponential line intensity distribution (Goody), and exponential tail with inverse line intensity distribution (Malkmus). The most widely applied statistical model is the exponential tail with inverse line intensity distribution, all of which are designed for Lorentzian-broadened spectral lines.

(11) $$P\left(S\right)\propto \left(\frac{1}{S}\right)exp\left(-\frac{S}{S_{M}}\right)$$

The average transmittance within the narrow spectral range Δ*η* can be calculated using the following formula:

(12) $${\bar{\tau }}_{\Delta \eta_{i}}\left(L\right)=exp\left\{-2a_{i}\left[{\left(1+\frac{{\bar{k}}_{\Delta \eta_{i}}X\left(L\right)}{a_{i}}\right)}^{1/2}-1\right]\right\}$$

In the equation, $a_{i}=\pi {\bar{b}}_{i}/{\bar{d}}_{i}$ is the spectral band fine-structure parameter, ${\bar{b}}_{i}$ is the average line width, ${\bar{d}}_{i}$ is the average line spacing; ${\bar{k}}_{\Delta \eta_{i}}$ is the average absorption coefficient within the spectral band; *X*(*L*) is the pressure-stroke length, which is extrapolated to standard conditions during use, i.e., *X*(*L*) = *PL*(296/*T*), where P and T represent the gas pressure and temperature, respectively.

Based on existing spectral line parameters within the spectral band, the three parameters above can also be determined theoretically. Young [37] provided a numerical averaging method for calculating spectral band model parameters, namely

(13) $${\bar{k}}_{\Delta \eta_{i}}=\frac{1}{\Delta \eta_{i}}\sum_{m=1}^{M}S_{i}^{m}$$

(14) $${\bar{b}}_{i}=\frac{1}{M}\sum_{m=1}^{M}b_{i}^{m}$$

(15) $${\bar{d}}_{i}=\frac{{\bar{k}}_{\Delta \eta_{i}}{\bar{b}}_{i}}{{\left(\frac{1}{\Delta \eta_{i}}\sum_{m=1}^{M}\sqrt{S_{i}^{m}b_{i}^{m}}\right)}^{2}}$$

In the equation, $S_{i}^{m}$ represents the intensity of the *m*th spectral line within the *i*th spectral region; $b_{i}^{m}$ denotes the half-width of the *m*th spectral line within the *i*th spectral region; *M* indicates the total number of spectral lines within the *i*th spectrum; $\Delta \eta_{i}$ defines the wavenumber range of the *i*th spectral region, cm^−1^. The required spectral parameters—line intensity and line half-width—must be referenced from the HITEMP database.

### 2.5. Infrared Radiation Transfer Model

#### 2.5.1. General Form

According to radiation transfer theory, when considering absorption, emission, and scattering within a medium, the radiation transfer equation can be expressed as follows:

(16) $$\frac{dI_{\eta }\left(s\right)}{ds}=-\sum_{j}k_{a\eta ,j}I_{\eta }\left(s\right)-\sum_{j}k_{s\eta ,j}I_{\eta }\left(s\right)+\sum_{j}k_{a\eta ,j}J_{\eta }\left(s\right)$$

In the equation, $I_{\eta }\left(s\right)$ and $J_{\eta }\left(s\right)$ represent spectral radiance and source function, respectively; $k_{a\eta ,j}$ and $k_{s\eta ,j}$ denote the absorption coefficient and scattering coefficient of the medium, respectively, with the subscript η=1/λ representing the wavenumber and *j* denoting the gas type. When an aero-engine operates in non-afterburner mode with complete combustion, the gas scattering effect can be neglected. Furthermore, if the medium is in local thermal equilibrium, based on Kirchhoff’s law and Planck’s law, the source function becomes the blackbody radiation function $I_{b\eta }$. Expressing the absorption coefficient as $k_{\eta ,j}$, the general form of the radiative transfer equation is

(17) $$\frac{dI_{\eta }\left(s\right)}{ds}=-\sum_{j}k_{\eta ,j}I_{\eta }\left(s\right)+\sum_{j}k_{\eta ,j}I_{b\eta }\left(s\right)$$

Solving the above differential form readily yields its integral form solution, namely

(18) $$\begin{array}{ll} I_{\eta }\left(L\right)= & \int_{0}^{L}\sum_{j}k_{\eta ,j}\left(s\right)I_{b\eta }\left(s\right)exp\left\{-\int_{0}^{L}\sum_{j}k_{\eta ,j}\left(s^{\;\prime }\right){ds}^{\;\prime }\right\}ds\\ & +I_{\eta }\left(0\right)exp\left\{-\int_{0}^{L}\sum_{j}k_{\eta ,j}\left(s\right)ds\right\} \end{array}$$

Among these, $I_{\eta }\left(L\right)$ is the spectral radiation measured at *s* = *L*, where the lower integration limit “0” denotes the boundary. $I_{\eta }\left(0\right)$ represents the external radiation contribution entering the transmission, corresponding to the blackbody radiation constant at ambient temperature.

#### 2.5.2. Narrow Band Radiation Transmission Equation

The exponent factor in Equation (18) can be replaced by spectral transmittance to simplify the expression of the radiative transfer equation (RTE).

(19) $$\tau_{\eta }\left(L\right)=exp\left\{-\int_{0}^{L}\sum_{j}k_{\eta ,j}\left(s\right)ds\right\}$$

Therefore, by deriving the transmittance, Equation (18) can be simplified into a concise form.

(20) $$I_{\eta }\left(L\right)=\int_{0}^{L}I_{b\eta }\left(s\right)\frac{d\tau_{\eta }\left(s\right)}{ds}ds+I_{\eta }\left(0\right)\tau_{\eta }\left(L\right)$$

In the spectral band model, the spectral range is subdivided into bands of width Δ*η*, where the wavenumber interval (5–25 cm^−1^) is sufficiently narrow to assume that the blackbody intensity remains essentially constant within each band, yet sufficiently broad to encompass a substantial number of absorption lines, enabling their statistical treatment. We obtained the narrow-band average transmittance.

(21) $${\bar{\tau }}_{\Delta \eta_{i}}\left(L\right)=\frac{1}{\Delta \eta_{i}}\int_{\eta -\Delta \eta_{i}/2}^{\eta +\Delta \eta_{i}/2}\tau_{\eta }\left(L\right)d\eta$$

Substituting (21) into (20) yields the narrow band formE.

(22) $${\bar{I}}_{\Delta \eta_{i}}\left(L\right)=\int_{0}^{L}{\bar{I}}_{b,\Delta \eta_{i}}\left(s\right)\frac{d\tau_{\Delta \eta_{i}}\left(s\right)}{ds}ds+{\bar{I}}_{\Delta \eta_{i}}\left(0\right){\bar{\tau }}_{\Delta \eta_{i}}\left(L\right)$$

## 3. Numerical Methods

### 3.1. Nozzle Parameter Solution Method

**Figure 2:**
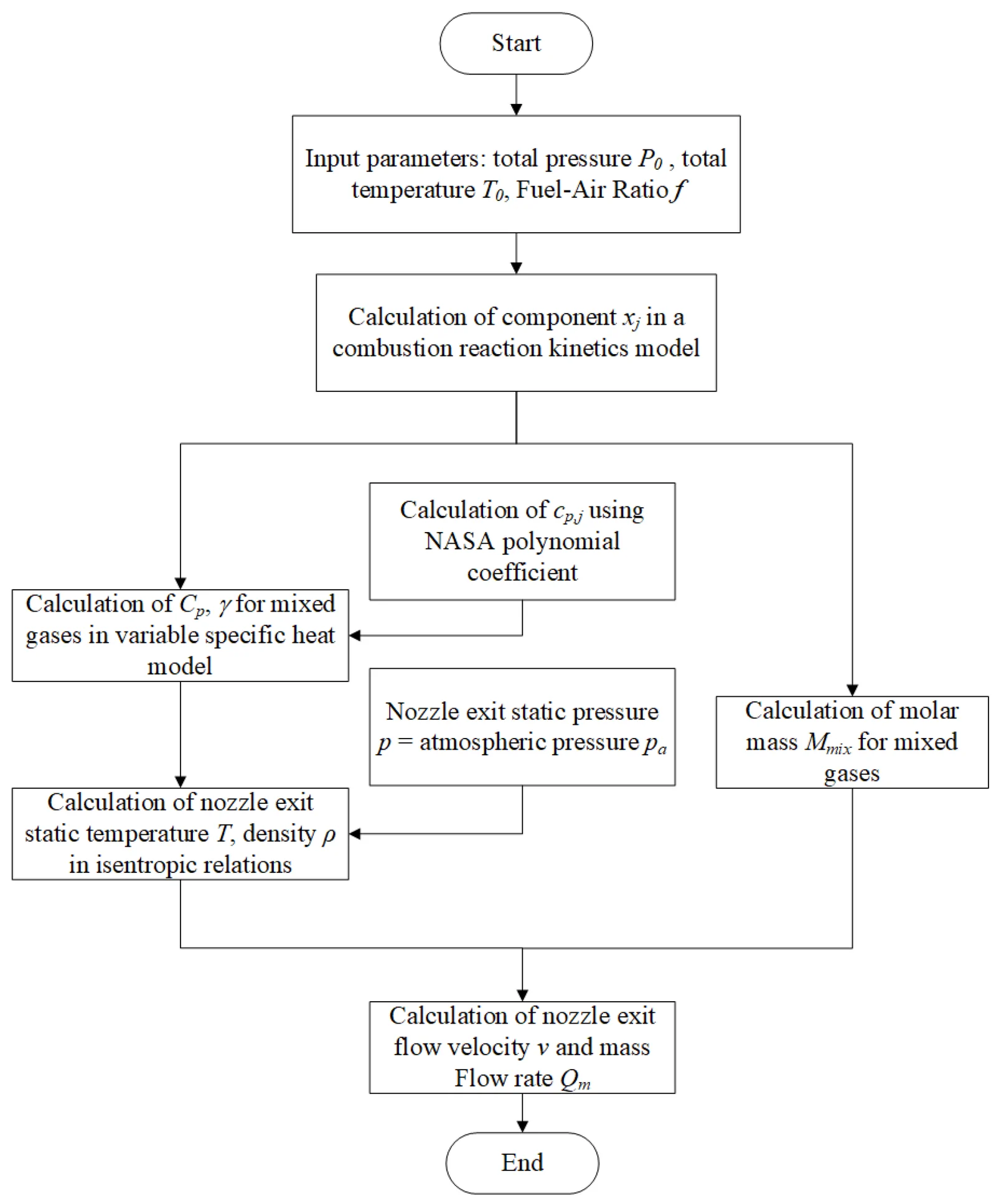
Nozzle parameter solution flowchart.

In this section, we describe the numerical methods applied for solving the nozzle parameters. The overall flowchart of nozzle parameter solution process is shown in Figure 2. Using the aforementioned combustion reaction kinetics model, based on the single-step chemical reaction between aviation kerosene (C_12_H_23_) and air, assuming the number of moles of fuel entering the aero-engine is *n_f_* and the number of moles of air participating in combustion is *n_a_*, with fuel combustion being sufficiently complete, the fuel–air ratio *f* can be expressed as follows:

(23) $$f=\frac{m_{f}}{m_{a}}=\frac{n_{f}}{n_{a}}\cdot \frac{M_{f}}{M_{a}}$$

Here, *m_f_* denotes the mass flow rate of fuel, *m_a_* denotes the mass flow rate of air, M_f_ represents the molar mass of fuel (167.304 g/mol), and *M_a_* represents the molar mass of air (28.97 g/mol). The fuel–air ratio for an aero-engine typically ranges from 0.025 to 0.031 [38].

The stoichiometric fuel–air ratio for C_12_H_23_/air combustion is approximately 0.068 (Equation (1)); the studied range 0.025–0.031 therefore corresponds to f/f_st_ = 0.37–0.45, i.e., fuel-lean combustion. Consistently, the O_2_ mole fraction in Table 1 remains positive (0.115–0.155) over the entire studied range, confirming oxygen-surplus (fuel-lean) operation and clearly defining the lean/rich boundary of the single-step model. The proportions of nitrogen and oxygen in the air are 0.79 and 0.21, respectively [39]. Under ideal conditions of complete combustion of aviation fuel (C_12_H_23_), the molar number and molar fraction of the hot exhaust jet gas can be expressed as shown in Table 1.

The ideal gas assumption posits that gas molecules exhibit no intermolecular forces and occupy no volume themselves. This idealized gas model describes the relationship among its macroscopic state parameters—static pressure *P*, density *ρ*, static temperature *T*, and molar mass of the mixture $M_{mix}$—via the following equation of state:

(24) $$P=\frac{\rho }{M_{mix}}RT$$

where R is the molar gas constant, taken as 8.314 $J/\left(mol\cdot K\right)$, and the molar mass of the mixture $M_{mix}=\sum_{j=1}^{n}x_{j}M_{j}$, specific heat ratio of mixture $\gamma =\frac{c_{p}}{c_{p}-R}$, specific heat capacity of a mixture $c_{p}=\sum x_{j}c_{pj}$, The specific heat capacity at constant pressure for each gas $c_{pj}$ is expressed as a polynomial function of temperature, employing the polynomial coefficients from NASA Technical Report [40], where $x_{j}$ is the molar fraction of component *j*, $\sum x_{j}=1$, $M_{j}$ is the molar mass of component *j* (g/mol). The mass flow rate *Q_m_* is the mass of fluid passing through the cross-sectional area per unit time (kg/s), which can be expressed as follows:

(25) $$Q_{m}=\rho S\nu$$

Here, *S* denotes the tail nozzle cross-sectional area, and *v* represents the exit exhaust gas velocity, which can be calculated using the isentropic expansion model [41]. The atmospheric inlet conditions are set to standard atmospheric conditions, assuming the expansion process within the nozzle is isentropic, with the exit static pressure *P* equal to the ambient atmospheric pressure.

The computational domain in this study begins at the nozzle exit plane. The molar fraction and mass flow rate at the nozzle exit plane were determined using the aforementioned computational method. When investigating the influence of the fuel–air ratio, the total temperature was set to 1000 K, and the fuel–air ratio was varied in increments of 0.002 to obtain values of 0.025, 0.027, 0.029, and 0.031. To investigate the effect of total temperature, the fuel–air ratio was fixed at 0.031, and the total temperature was varied in 200 K increments to 400 K, 600 K, 800 K, and 1000 K. The total pressure was set to a constant value of 303.975 kPa.

### 3.2. Methods for Solving Wake Flow Field

This study employs the Finite Volume Method (FVM) to solve fluid control equations, utilizing a time-stepping approach to obtain steady-state solutions. All computations were conducted on structured meshes comprising 200,000 and 220,000 elements, verifying the solution’s mesh independence. Spatial discretization employed a second-order accurate scheme, where diffusion flux was discretized using central differencing and advection flux was handled via the upwind-biased form. Standard wall functions were applied near wall boundaries. For the inviscid flux vector, a high-order discretization technique based on the Total Variation Decrease (TVD) principle was adopted. Turbulence was set at 2%, with a turbulence length scale of 0.001 m.

### 3.3. Methods for Solving the Radiative Transfer Equation

The analytical solution of the radiative transfer equation is highly complex. Currently, numerical computation is widely employed to solve radiative transfer problems in engine wakes. Since engine wakes consist of gases and the influence of scattering media on radiative transfer is negligible, LOS [42] can be used to solve the radiative transfer equation. Its fundamental principle simplifies radiative transfer in three-dimensional non-uniform media to one-dimensional multi-layer media. Each LOS is discretized into m layers that coincide with the CFD grid cells intersected by the LOS, with the layer thickness ranging from 0.5 mm near the nozzle exit to 5 mm in the far field. A layer is treated as a homogeneous, isothermal medium when the intra-layer temperature variation is less than 1% of the local temperature and the species mole-fraction variation is less than 1%; this criterion is automatically satisfied in the near-nozzle region due to the fine grid and was verified a posteriori for the far-field cells. A line of sight parallel to the detector direction penetrates the entire wake computational domain. Each line of sight is divided into m layers. Assuming each layer contains a homogeneous, isothermal medium, the spectral distribution with a resolution of 2 cm^−1^ is computed at each grid point along the LOS line in the reverse direction. Its discrete form is expressed as follows:

(26) $$I_{\Delta \eta_{i}}^{k}=I_{\Delta \eta_{i}}^{k-1}\tau_{\Delta \eta_{i}}^{k}+I_{b,\Delta \eta_{i}}^{k}\left(1-\tau_{\Delta \eta_{i}}^{k}\right)$$

**Figure 3:**
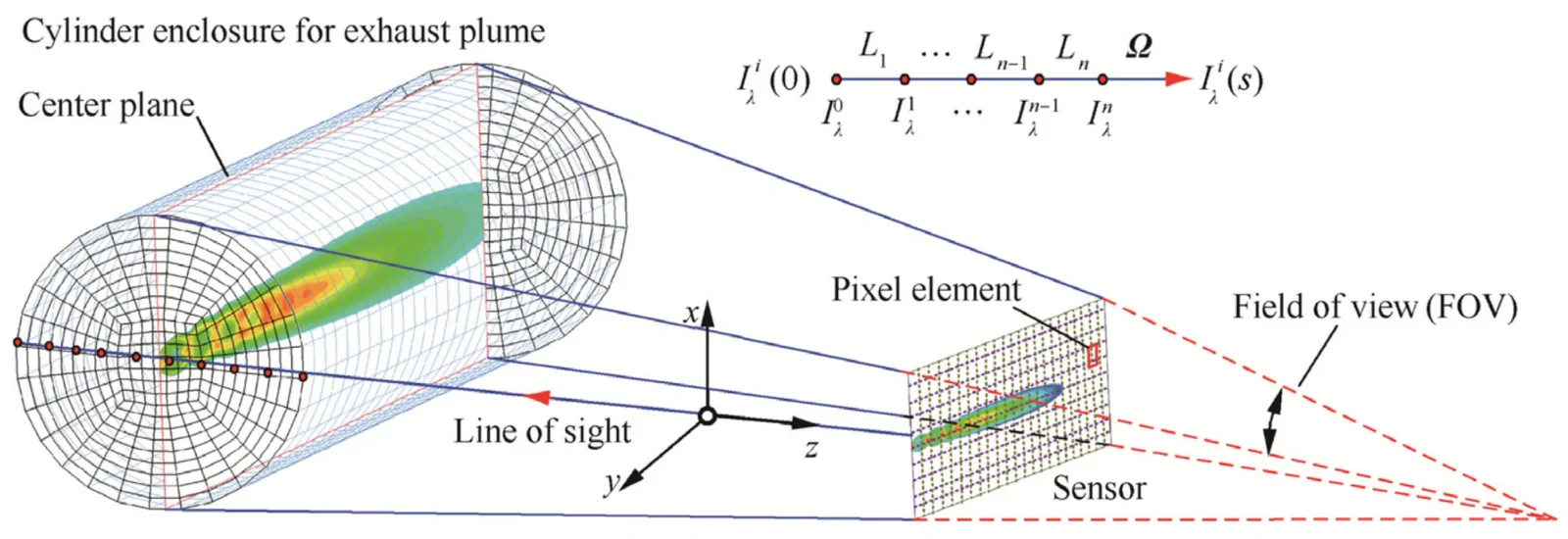
Flowchart of the narrow-band LOS radiation transfer solution.

Here, $I_{\Delta \eta_{i}}^{k}$ and $\tau_{\Delta \eta_{i}}^{k}$ denote the radiation intensity and transmittance at the *k*th layer of the medium with spectral band length $\Delta \eta_{i}$. The recursive solution procedure is summarized as follows: (i) input the flow-field data (*T*, *P*, *x_j_*) obtained from the k-ε simulation; (ii) define the LOS grid according to the detector geometry; (iii) compute the SNB parameters ($\bar{k}$,$\;\bar{b}$,$\;\bar{d}$) from the HITEMP database using Equations (13)–(15); (iv) compute the layer transmittance using the Malkmus model (Equation (12)); (v) march along the LOS in the reverse direction, applying the recursive discrete form (Equation (26)) layer by layer; and (vi) output the spectral radiation intensity at a resolution of 2 cm^−1^. The flowchart of LOS radiation transfer solution is shown in Figure 3.

### 3.4. Numerical Method Validation

**Figure 4:**
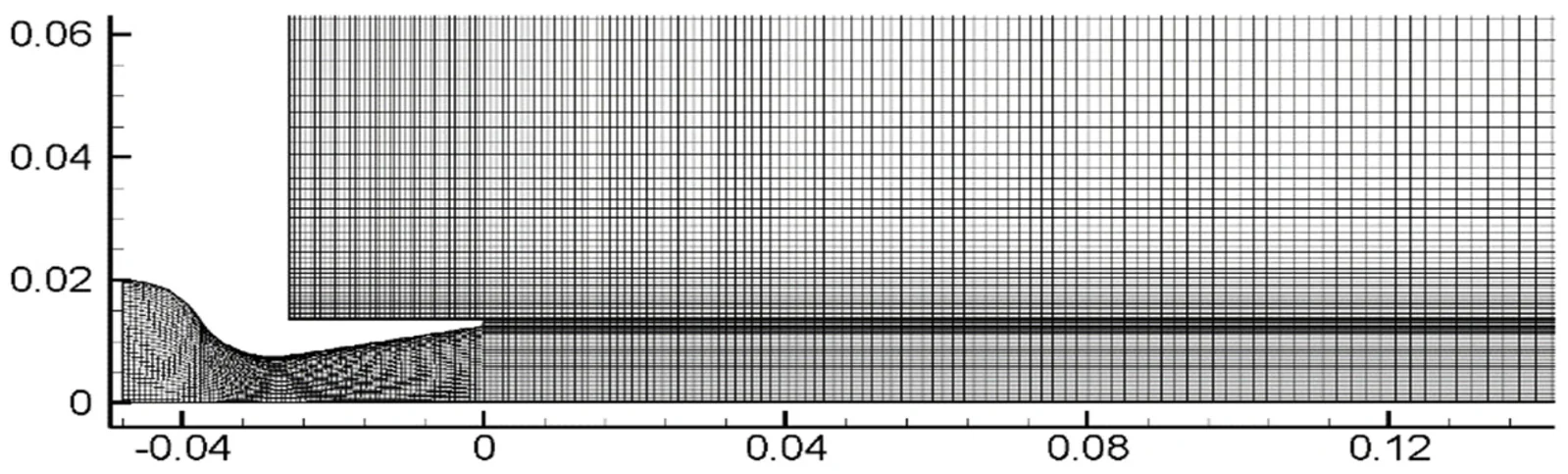
Computational regional modeling.

In 2001, the Advanced Research and Development Center, where Avital [35] worked, conducted ground-based infrared radiation measurements on the BEM-2 engine. This engine featured a nozzle diameter of 25 mm and utilized an AP/HTPB propellant formulation without aluminum powder additives. During measurements, both the spectrometer and imager were positioned 9.4 m from the axial cross-section of the exhaust plume. Both instruments were calibrated using a blackbody, and radiation measurements were corrected by subtracting background grayscale values. The measurements ultimately yielded infrared radiation intensity distributions within the 1.372–1.516 μm wavelength band and spectral intensity curves within the 1.5–5.5 μm band. Given the propellant formulation, nozzle dimensions, and gas composition at the nozzle exit, an appropriate computational model must be selected for validation. The structure and boundary conditions of the computational domain are shown in Figure 4.

Selection of nozzle calculation parameters, including temperature, pressure, Mach number, and gas composition fractions, is shown in Table 2.

**Figure 5:**
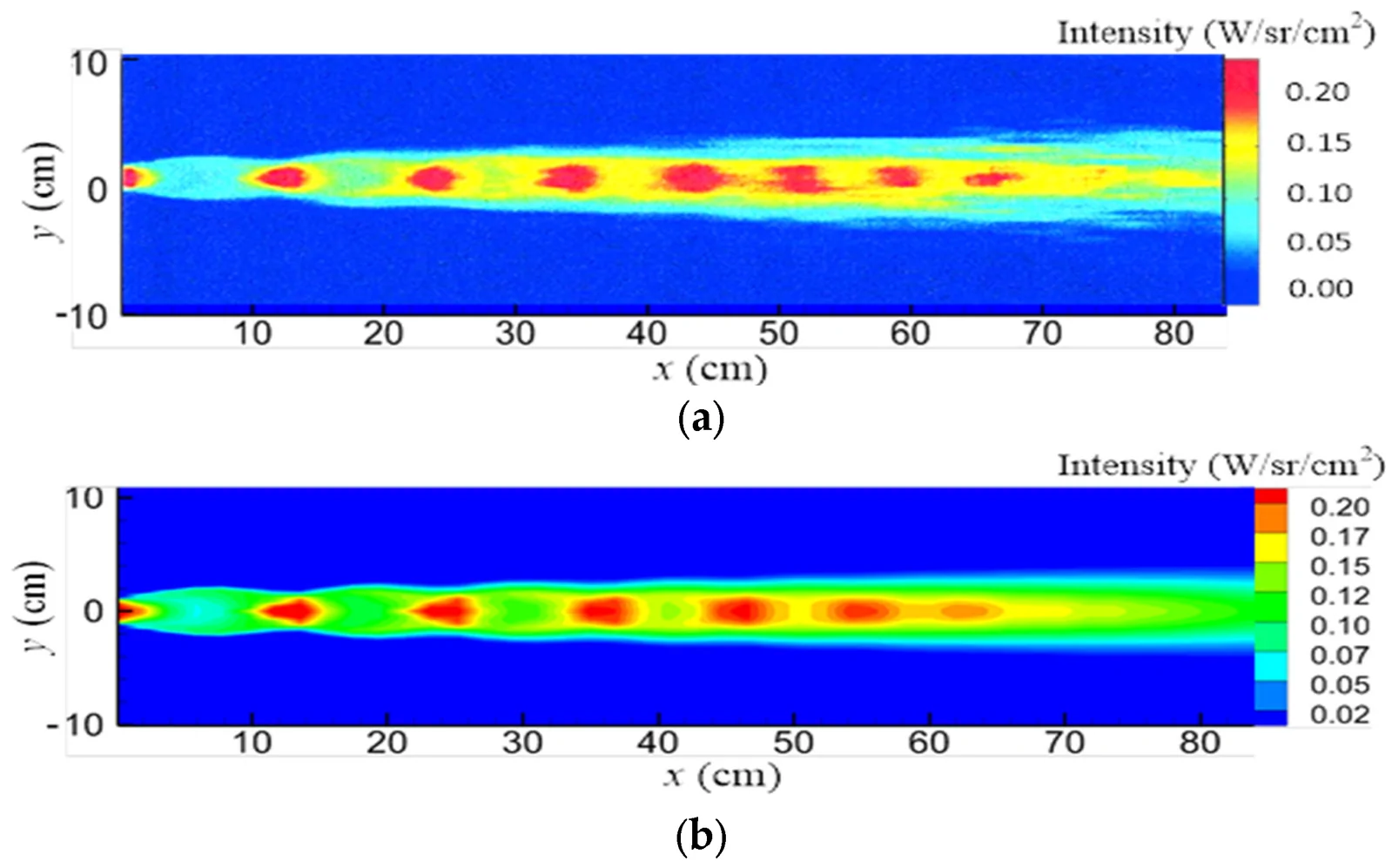
Comparison of computational results with Israeli BEM experimental findings [35]. (**a**) Radiation intensity map obtained from experiments in the 1.372–1.516 μm wavelength band. (**b**) Radiation intensity map calculated for the 1.372–1.516 μm wavelength band.

**Figure 6:**
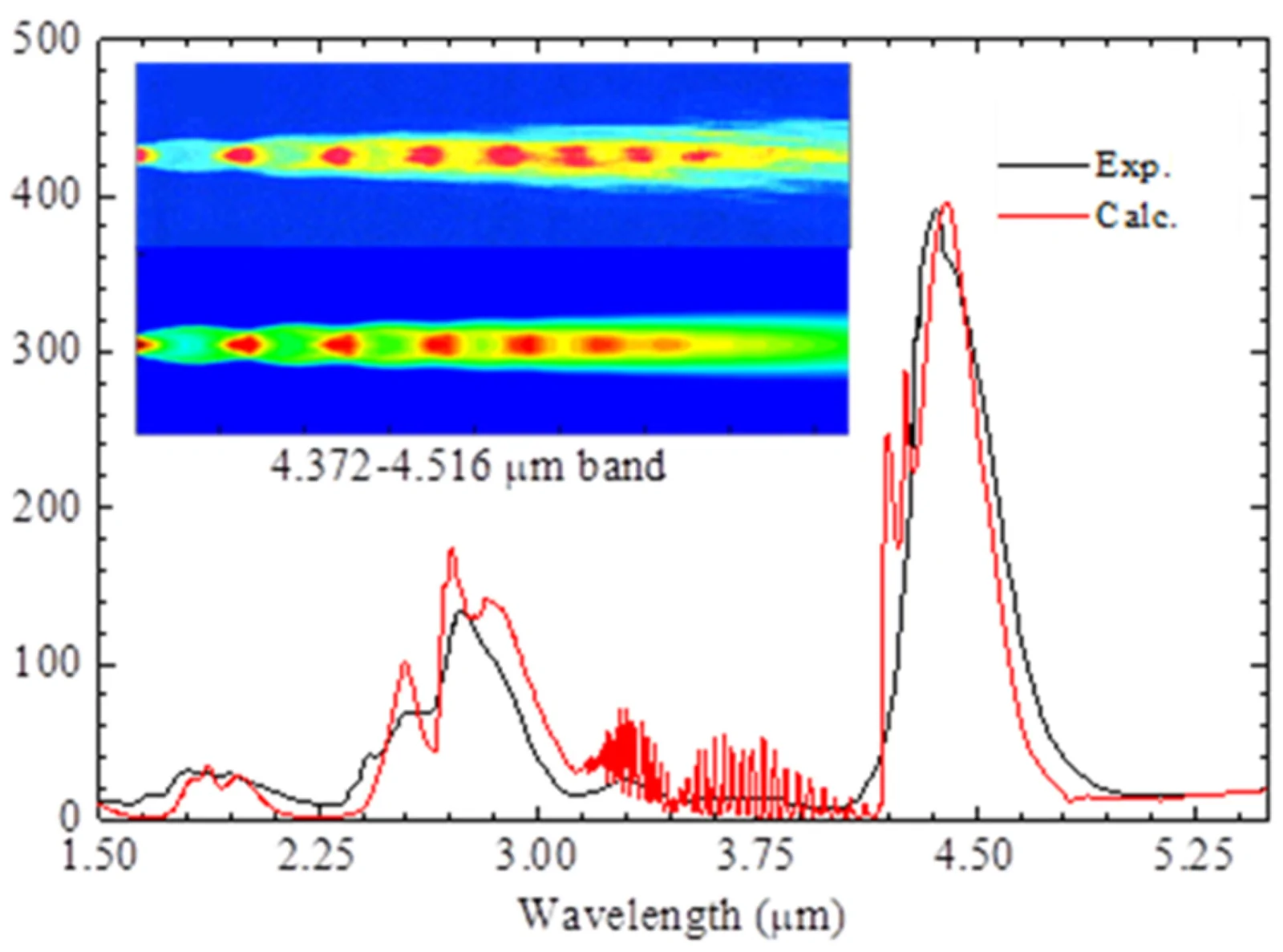
Comparison of computed radiation intensity with experimental measurements [35].

Figure 5 presents a comparison between experimentally measured and computationally derived infrared radiation intensity distributions within the 1.372–1.516 μm wavelength band. The number of Mach node locations captured in the computational image, their corresponding positions, and radiation intensities all exhibit good agreement with measured values. Figure 6 displays the comparison results between experimentally measured and computationally derived spectral radiation intensity curves within the 1–6 μm wavelength range. As shown, the error range between predicted and measured values is controlled within 10%. Detailed data are provided in Table 3.

It should be noted that the BEM-2 engine employs AP/HTPB solid propellant, whose exhaust products include species such as HCl that are absent from RP-3 kerosene combustion, where CO_2_ and H_2_O are the dominant infrared-active species. Therefore, while the present validation demonstrates the model’s capability to handle high-temperature underexpanded jets in a general sense, it does not directly validate the single-step combustion model and the corresponding radiative properties specifically for RP-3 kerosene. A sensitivity analysis of the CO_2_/H_2_O concentrations was performed to confirm that the simplified single-step model yields reasonable spectral radiative intensity predictions under the operating conditions considered, with the results indicating that concentration variations within the fuel–air ratio range of 0.025–0.031 produce proportional changes in peak intensities without altering the fundamental spectral structure. In addition, a sensitivity analysis of intermediate combustion products was performed by injecting small CO mole fractions (0.1–1%, representative of fuel-lean combustion equilibrium at the nozzle exit) into the SNB–LOS calculation and quantifying the resulting variation in the peak intensity at 2143 cm^−1^ (CO fundamental band) and 2349 cm^−1^ (CO_2_ band). The results show that, within the studied fuel-lean regime, the contribution of intermediate products is below 2% of the total band-integrated intensity, confirming that the single-step model is adequate for the operating range considered.

### 3.5. Atmospheric Absorption Effects of Wake Spectra in the Infrared Band

Atmospheric absorption in the infrared spectrum refers to the phenomenon where primary atmospheric gases absorb infrared radiation, thereby attenuating radiant energy. Water vapor (H_2_O) constitutes a significant proportion of the atmosphere, with its concentration varying substantially across regions and weather conditions. In humid environments, its absorption of infrared radiation from exhaust plumes becomes more pronounced. Multiple absorption bands exist in the near- and mid-infrared regions, such as the O-H stretching vibration centered near 3756 cm^−1^ and the bending vibration of the H_2_O molecule centered around 1590 cm^−1^. Atmospheric CO_2_ levels are relatively stable, with its primary absorption band centered near 2349 cm^−1^, caused by its asymmetric stretching vibration. O_3_ molecules in the atmosphere are primarily concentrated in the ozone layer and have a relatively minor impact on aircraft wake spectrum absorption compared to H_2_O and CO_2_, with its main absorption band centered near 1041 cm^−1^.

Our team conducted measurement experiments on the spectra of multiple aircraft types’ wakes using an FTIR. We analyzed ground-based aircraft wake spectra at different detection distances from a ground-based platform and numerically simulated atmospheric transmittance under various atmospheric models from a space-based platform. This research investigates the absorption effects of the atmosphere on wake spectra under different scenarios.

#### 3.5.1. Experimental Data (Ground-Based Platform Detecting Surface Aircraft)

**Figure 7:**
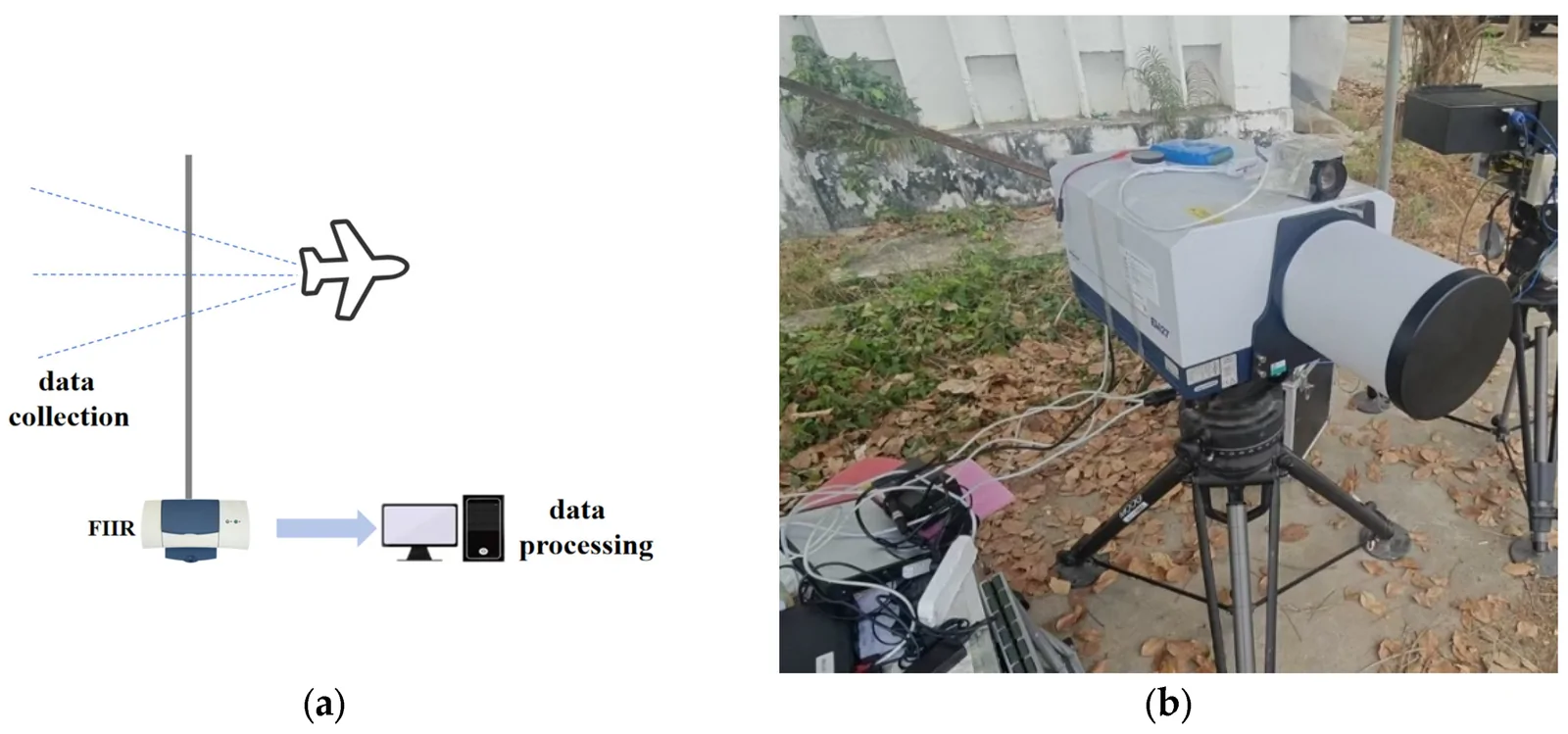
Data acquisition. (**a**) Schematic diagram of the field experiment scene; (**b**) Picture of the experimental site.

Infrared spectroscopy measurements were performed using an EM27 FTIR (Bruker Corporation). The spectrometer covers a measurement range of 2.5–12 μm, with a spectral resolution of 0.5 cm^−1^, wavenumber accuracy of 0.05 cm^−1^ @ 2000 cm^−1^, operating in passive mode with 8 stacked scans. Research is currently underway in the 2–15 μm wavelength range with a resolution exceeding 0.25 cm^−1^. Within the studied field of view (FOV) of 30 mrad, the focal length is 2000 mm, the optical aperture is 1000 mm, and the noise-equivalent spectral radiance is 6 × 10^−8^ W·cm^−2^·sr^−1^·cm^−1^. The field experiment scene for data acquisition is shown in Figure 7.

**Figure 8:**
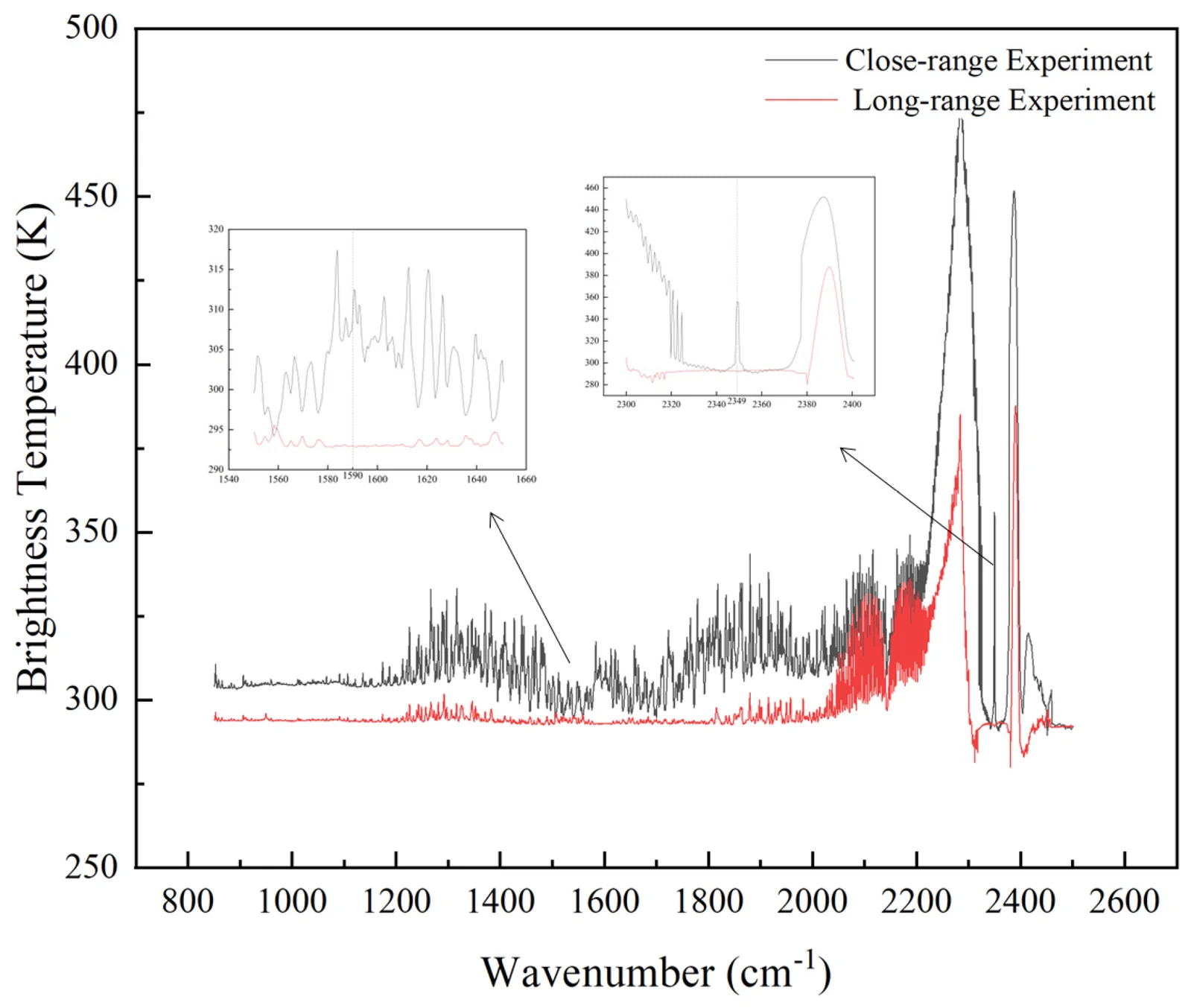
Comparison of experimental data at near and far distances.

During close-range testing, the instrument was positioned 5 m from the wake. For long-range testing, it was placed 400 m away. The field-of-view axis was perpendicular to the wake axis to maximize received radiation intensity. Ambient conditions were 18 °C and 78% relative humidity. Instrument calibration was performed using a blackbody. Experimental data is presented in Figure 8.

#### 3.5.2. Simulated Data (Space-Based Platform Detecting Aircraft in Flight)

Researchers, including Wei Heli from the Institute of Optics and Precision Mechanics, Chinese Academy of Sciences, developed the Combined Atmospheric Radiative Transfer (CART) software [43,44,45,46], which incorporates atmospheric parameters for typical regions across China. Compared to international models like MODTRAN, CART integrates parameter profiles from numerous domestic locations, enabling more accurate simulation of atmospheric transmission patterns across various Chinese regions. It employs line-by-line integration and parameterized atmospheric molecular concentrations to calculate the mean atmospheric transmittance within a 0.1 cm^−1^ bandwidth. The mean atmospheric transmittance is modeled using a fourth-order nonlinear exponential equation

(27) $${\bar{T}}_{v}\left(t,p,u\right)=e^{\left\{-u\cdot e^{\left(\sum_{i=0}^{4}c_{i}\left(t,p\right)\left[logu^{t}\right]\right)}\right\}}$$

where ${\bar{T}}_{v}\left(t,p,u\right)$ denotes the average transmittance of a given atmospheric molecule at a specific wavenumber *v* ± 0.5 cm^−1^ under atmospheric temperature *t*, atmospheric pressure *p*, and molecular absorption content *u*; *c_i_*(*t*,*p*) represents the fitting coefficients corresponding to *i* = 0, 1, 2, 3, 4.

**Figure 9:**
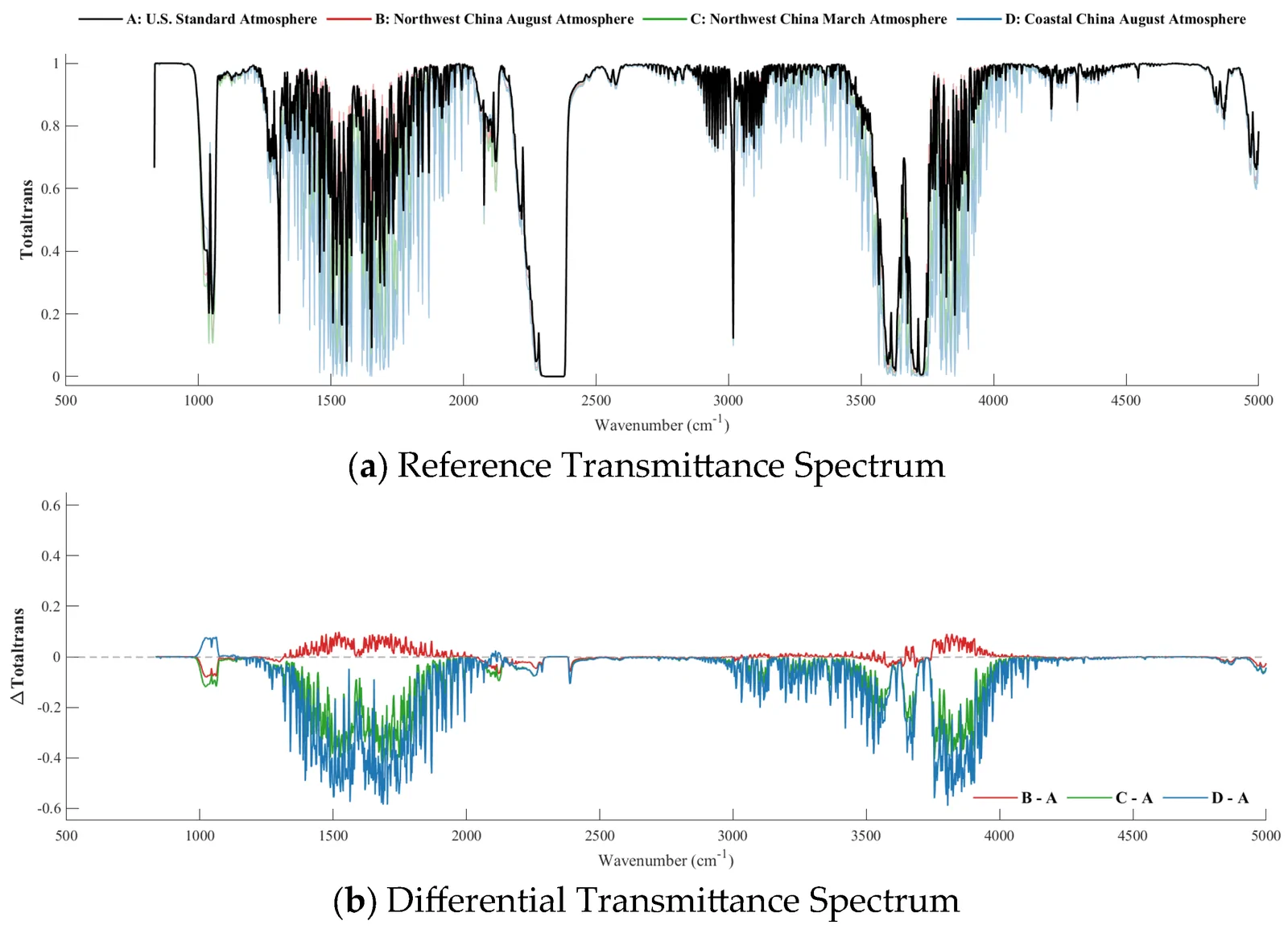
Atmospheric spectral transmittance.

The energy radiated from aircraft wakes undergoes atmospheric absorption and attenuation during its propagation to space-based platform detectors. The selected conditions are the U.S. Standard Atmosphere model, the March atmospheric model for the Northwest region, the August atmospheric model for the Northwest region, and the August atmospheric model for coastal areas; observation altitude: 500 km; target altitude: 10 km; observation zenith angle: 135 degrees; wavelength range: 833 cm^−1^ to 5000 cm^−1^ (2 μm to 12 μm); spectral resolution: 2 cm^−1^. The atmospheric spectral transmittance is shown in Figure 9 below.

## 4. Results and Discussion

This study investigates the wake field of an aero-engine, focusing on three key factors: fuel–air ratio, exhaust temperature, and atmospheric absorption effects. Through numerical simulation, it analyzes the distribution of temperature, Mach number, and characteristic gas (CO_2_ and H_2_O) mole fractions within the wake field, while examining factors influencing infrared spectral features. The flow field simulations are based on the Reynolds-Averaged Navier–Stokes (RANS) equations coupled with the k-ε turbulence model. Spectral calculations employ the SNB model integrated with the LOS. All simulation results undergo independence verification to ensure data reliability. The following sections systematically analyze the influence mechanisms of fuel–air ratio, exhaust temperature, and atmospheric absorption effects on the flow characteristics of the wake field and infrared spectral features across these three dimensions.

### 4.1. Influence of Fuel–Air Ratio

**Figure 10:**
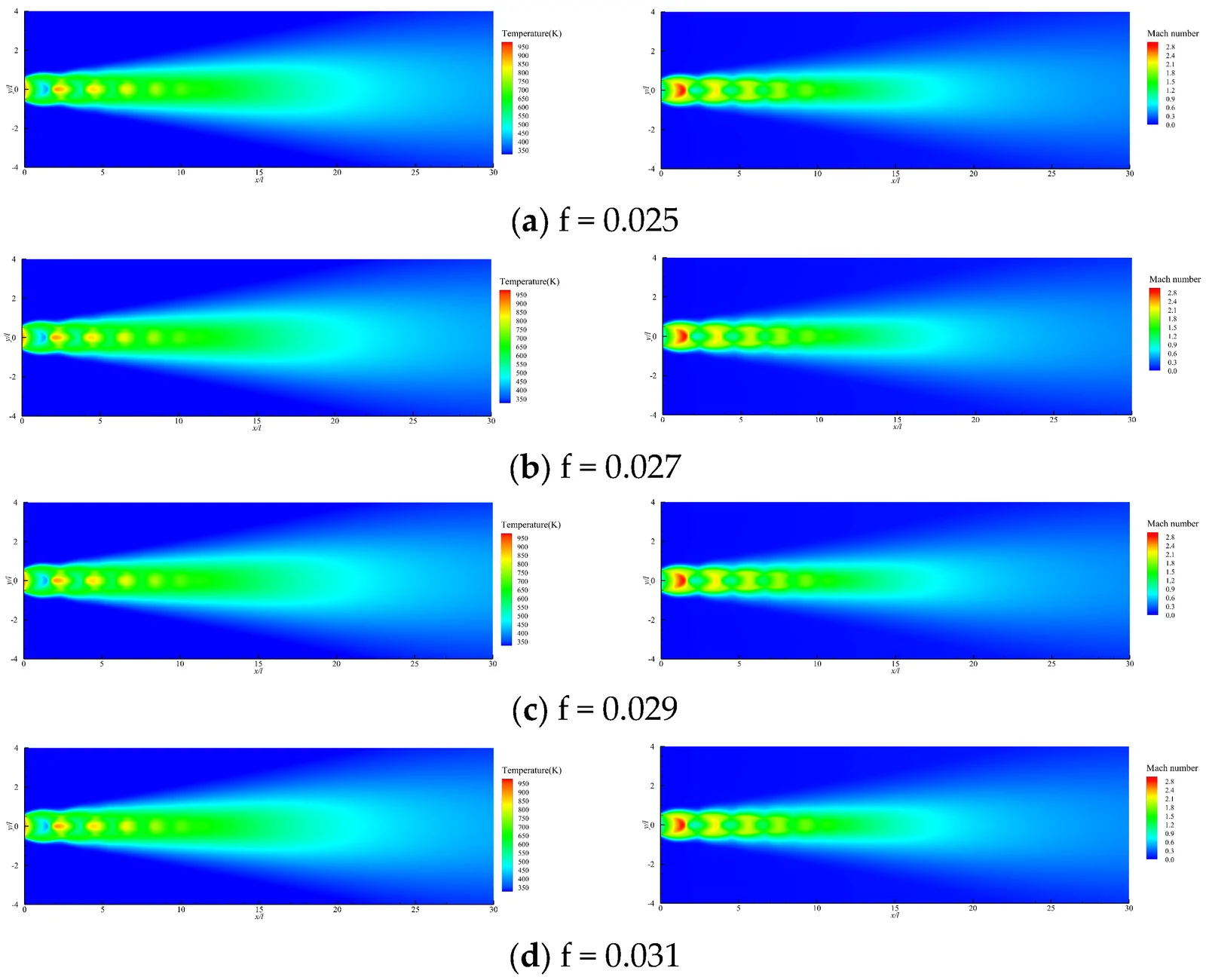
Temperature and Mach number distribution.

The temperature distribution (Figure 10) reveals a distinct “stable core-attenuating periphery” characteristic in the wake field across different fuel–air ratios. Near the nozzle (x/l ≤ 5), the core region maintains temperatures between 750 and 950 K. As the flow develops axially (5 < x/l ≤ 30), temperatures exhibit a gradient decay across all fuel–air ratio conditions, dropping to 420 K at x/l, representing a 46.2% decrease from the core region.

Regarding the Mach number distribution (Figure 10), the influence of the fuel–air ratio primarily manifests in the expansion extent of the wake: under high fuel–air ratio conditions (f = 0.031), the peak Mach number occurs at x/l = 3 with a small expansion angle (approximately 12°). Under low fuel–air ratio conditions, the expansion angle increases to 15°. This difference stems from the fact that under fully combusted conditions, a lower fuel–air ratio reduces the proportion of water and carbon dioxide in the combustion products, decreasing the gas density. According to the gas equation of state, this results in more pronounced fluid expansion under the same pressure gradient, leading to a broader expansion range. Furthermore, the Mach number stabilizes at x/l ≥ 20 across all operating conditions, indicating that the wake has fully mixed with the ambient air at this point, and the flow state has entered the subsonic turbulent region.

**Figure 11:**
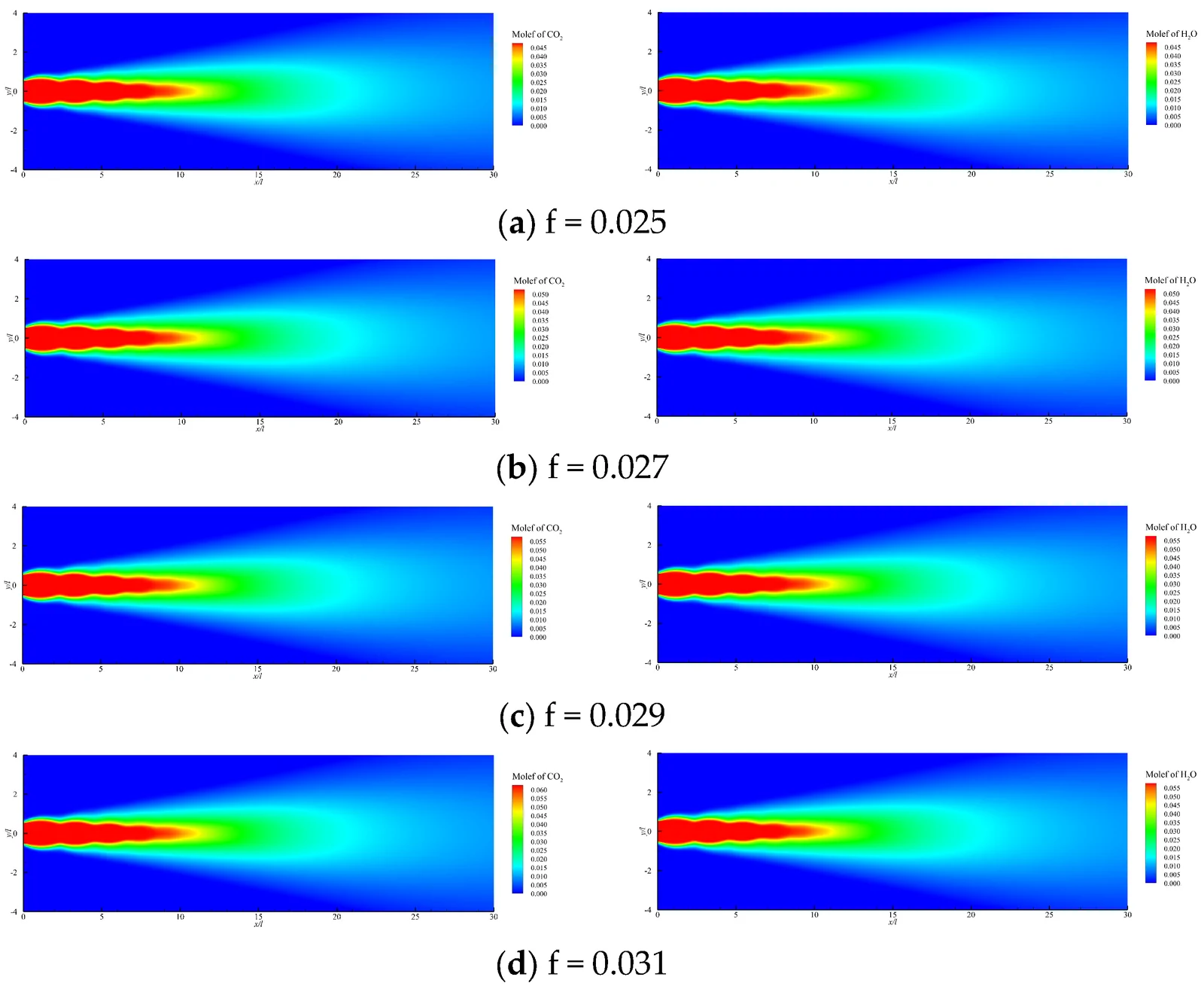
Molar fraction distribution of characteristic gases.

From the distribution of characteristic gas molar fractions (Figure 11), CO_2_ and H_2_O serve as the primary infrared radiation sources in the wake field. Their molar fraction distributions directly influence infrared spectral radiation intensity, both exhibiting an increasing trend with rising fuel–air ratio. Furthermore, both show a rapid decline when x/l ≥ 12 and y/l ≥ 1. This is attributed to the diluting effect of ambient air—where inert atmospheric components like N_2_ and O_2_ mix with the wake, reducing the relative concentration of characteristic gases. The dilution rate accelerates with increasing wake expansion angle, consistent with the Mach number distribution pattern.

**Figure 12:**
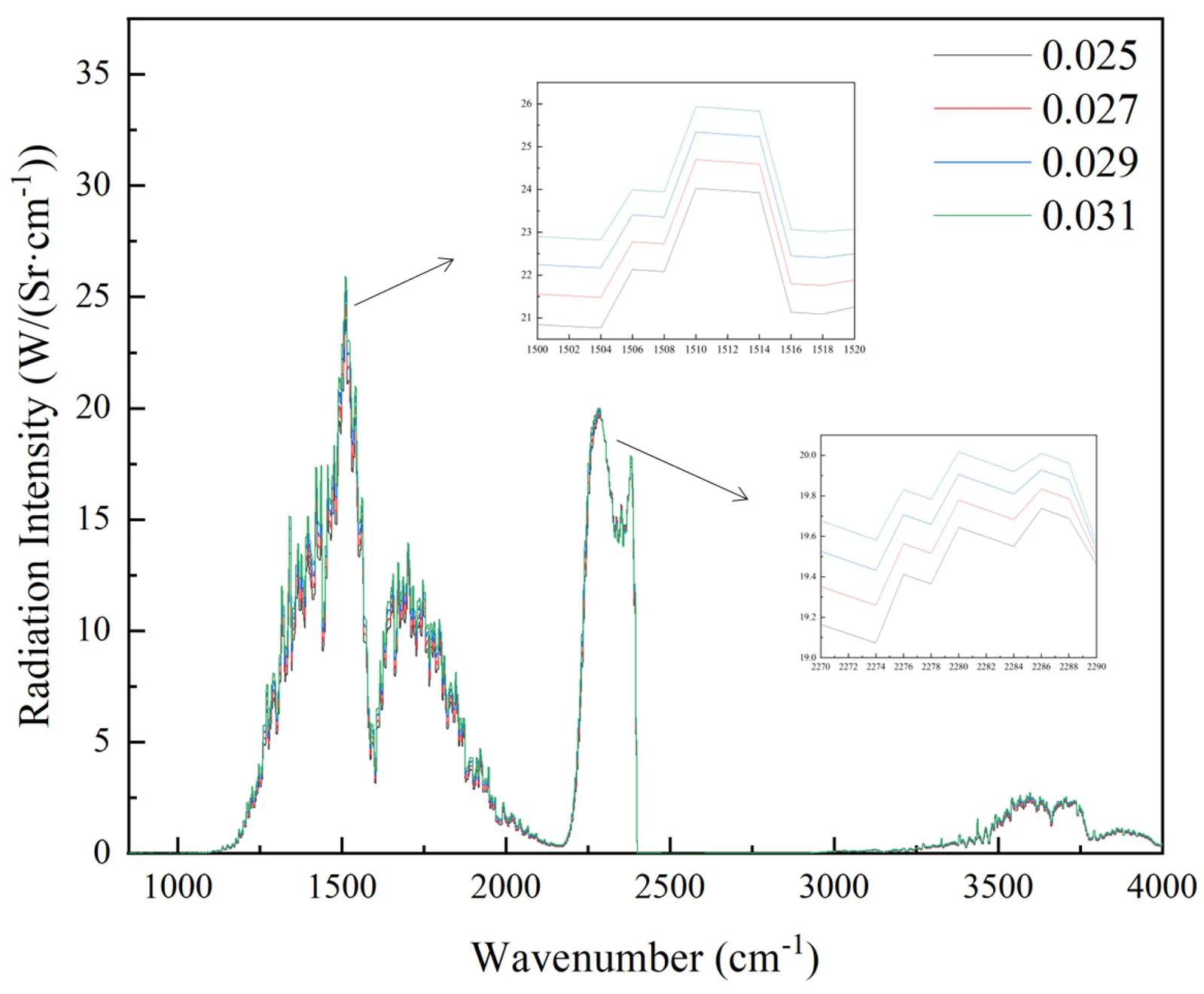
Influence patterns of fuel–air ratio on infrared spectral features.

The spectral band range spans 833 cm^−1^ to 4000 cm^−1^ (2.5 μm to 12 μm). From the perspective of infrared spectral features (Figure 12), the fuel–air ratio directly modulates spectral peak intensity and full width at half maximum (FWHM) by altering the concentration of characteristic gases in the wake. At high fuel–air ratios, increased production of CO_2_ and H_2_O molecules during combustion leads to a higher total number of transition molecules, boosting the probability of vibrational–rotational transitions. Combined with the Doppler broadening effect, this expands the FWHM while the peak increases in intensity but becomes flatter. These conclusions hold within the fuel-lean operating regime (f = 0.025–0.031, f/f_st_ ≤ 0.45).

### 4.2. Influence of Exhaust Temperature

The exhaust temperature, as the initial thermodynamic condition of the wake field, directly influences the fluid’s density, viscosity, and other physical parameters, thereby altering the flow structure and component diffusion characteristics within the wake field. This study selected four sets of exhaust temperatures to simulate the evolution patterns of the wake field under different engine operating conditions, focusing on analyzing the influence of temperature on flow characteristics, component distribution, and infrared spectral features.

**Figure 13:**
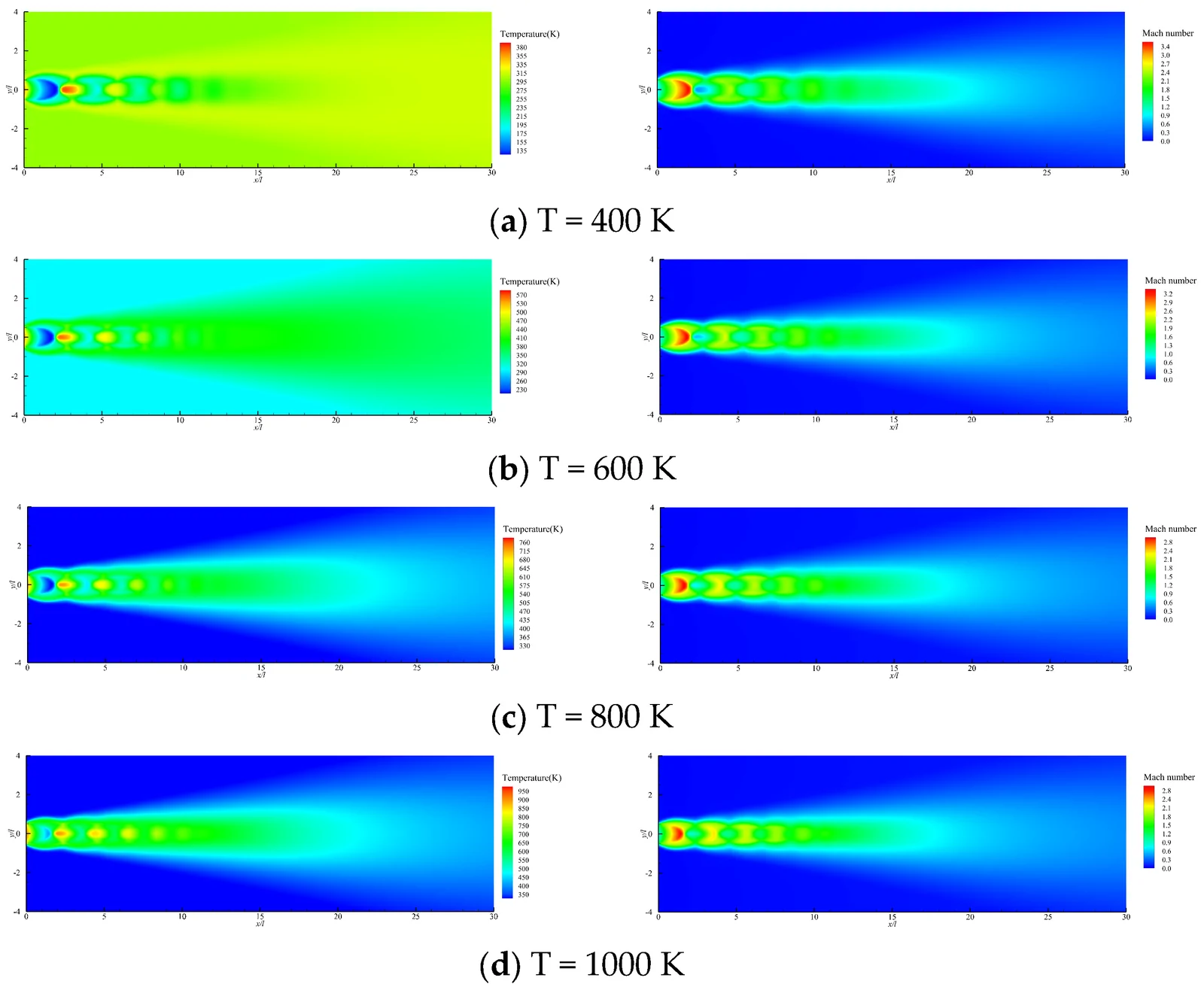
Temperature and Mach number distribution.

From the temperature distribution (Figure 13), it is evident that the exhaust temperature exerts a “global amplification” effect on the temperature distribution of the wake field: near the nozzle exit, the temperature in the wake core exhibits a linear positive correlation with the exhaust temperature—as T_0_ increases from 400 K to 1000 K, the core temperature rises from 380 K to 950 K, indicating nearly 100% heat transfer efficiency from the exhaust temperature. During axial development, higher exhaust temperatures result in slower temperature decay rates. This occurs because the fluid kinetic energy is greater in high–exhaust-temperature wakes. When mixing with ambient air, stronger turbulent pulsations enhance heat transfer and reduce local heat losses.

From the Mach number distribution (Figure 13), with a fixed exit velocity, according to the ideal gas equation of state, an increase in exhaust temperature directly reduces the density of the outlet fluid. According to the law of mass conservation, this density reduction decreases the mass flow rate through the nozzle per unit time. Consequently, the “momentum reserve” available for acceleration during axial development diminishes—fluids with lower mass flow rates are more susceptible to ambient air resistance, making it difficult to achieve high Mach numbers. This ultimately manifests as a reduction in the maximum axial Mach number.

**Figure 14:**
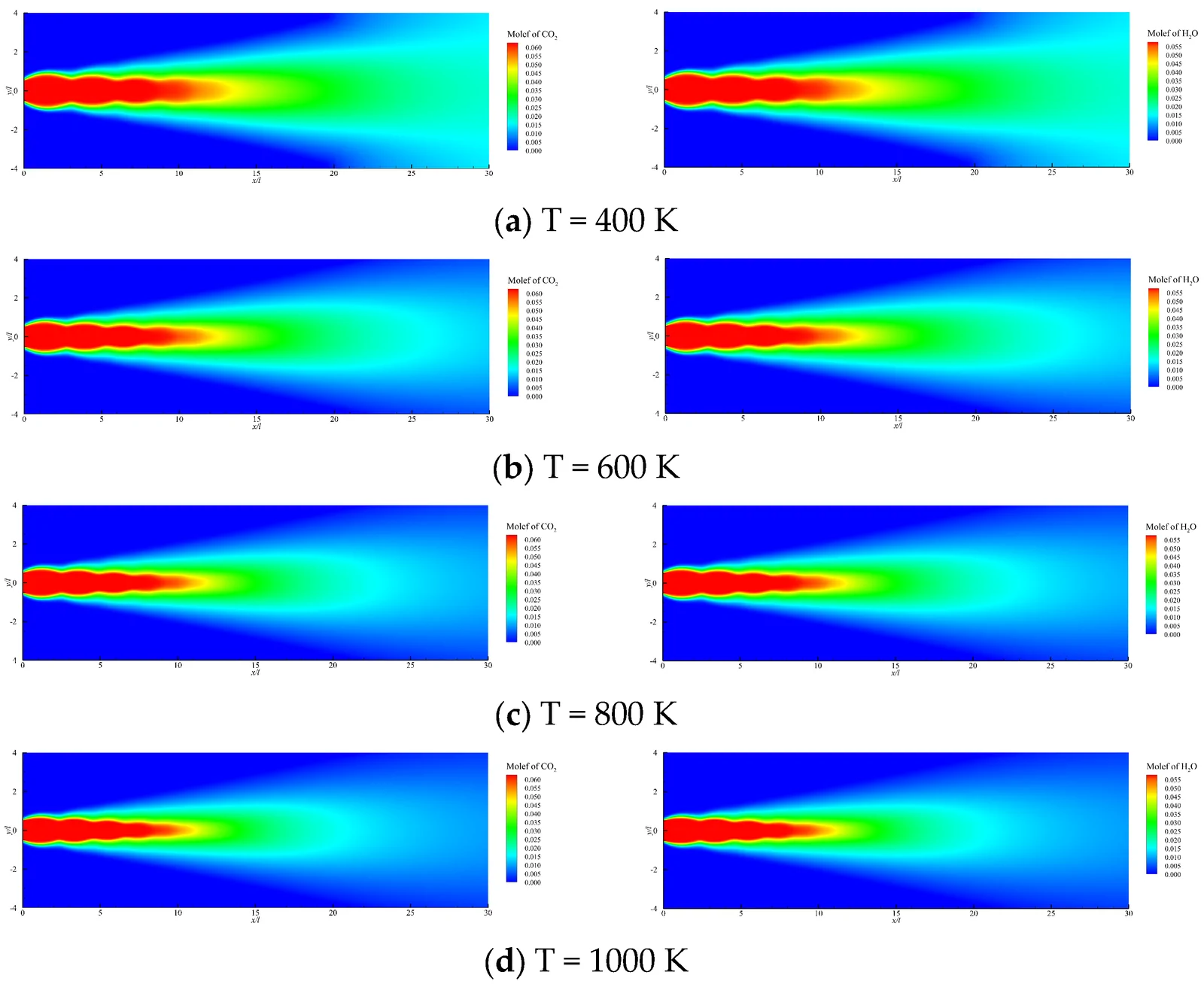
Molar fraction distribution of characteristic gases.

From the distribution of characteristic gas molar fractions (Figure 14), it is evident that under fixed outlet velocity at the tail nozzle and constant fuel–air ratio, the exhaust temperature exerts a negligible influence on the molar fraction distribution of CO_2_ and H_2_O. This phenomenon fundamentally stems from the combined effects of “combustion chemical equilibrium determining the total production” and “diffusion dilution dominating the decay process”: when fuel composition and fuel–air ratio are fixed, the initial production of CO_2_ and H_2_O remains constant. The dominant diffusion dilution in the tail flow further counteracts the indirect effects of temperature on reaction and diffusion rates, ultimately resulting in essentially unchanged molar fractions. This conclusion provides a clear direction for tail flow composition control: to alter CO_2_ and H_2_O molar fractions, prioritize adjusting the fuel–air ratio or fuel composition rather than relying on temperature regulation.

**Figure 15:**
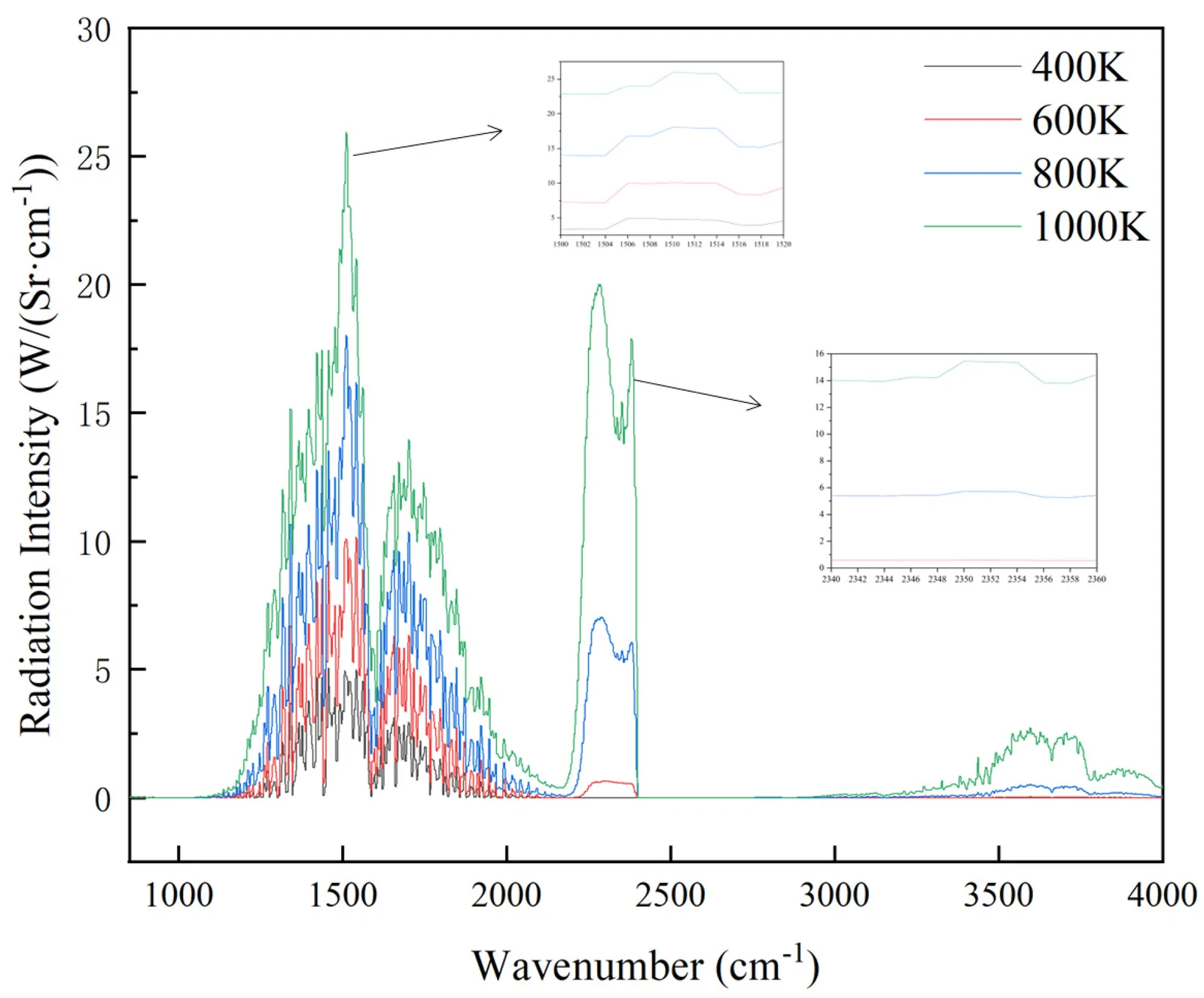
Influence patterns of exhaust temperature on infrared spectral features.

From the perspective of infrared spectral features (Figure 15), the influence of the exhaust temperature on the wake spectrum exhibits a “band-selective enhancement-global structural regulation” pattern. As the exhaust temperature increases from 400 K to 1000 K, the spectral response near the two major core emission bands at 1590 cm^−1^ (H_2_O bending vibration) and 2349 cm^−1^ (CO_2_ antisymmetric stretching vibration) becomes most pronounced, with no new characteristic emission bands emerging; The overall spectral intensity exhibits exponential growth. When the exhaust temperature increases from 400 K to 1000 K, the peak intensity at 1510 cm^−1^ rises from 4.75 to 25.94, representing a 446% increase. This phenomenon fundamentally stems from temperature-induced alterations in the population distribution of H_2_O molecular vibrational–rotational energy levels. According to the Boltzmann distribution law, the proportion of high-energy-level populations exhibits exponential growth with increasing temperature, leading to a significant enhancement in transition probability. Zhu et al. [6] also confirmed in their study of H_2_O spectra in rocket exhaust plumes that “a 200 K temperature increase boosts radiation transition probability in H_2_O characteristic bands by 30–50%,” consistent with the 30% peak intensity increase observed in this study when temperature rises from 800 K to 1000 K. H_2_O peak redshifts, with the redshift rate accelerating at higher temperatures. This occurs because molecular vibrational energy level spacing narrows at elevated temperatures, increasing the proportion of low-energy transitions. CO_2_ peak positions shift minimally, as its energy level spacing is temperature-insensitive. The broadening effect intensifies: rising temperatures expand the Doppler broadening of CO_2_ and H_2_O half-widths while exciting higher-order H_2_O transitions, enriching spectral fine structure.

### 4.3. Influence of Atmospheric Absorption Effect

Based on the experimental data (Figure 8), the spectral horizontal axis ranges from 833 cm^−1^ to 2500 cm^−1^ (4–12 μm), while the vertical axis represents brightness temperature. This wavelength range was selected for analysis because solar radiation approximates that of a 5800 K blackbody, with a peak wavelength around 0.5 μm. The energy is primarily concentrated in the ultraviolet–visible–near-infrared band (0.2–3 μm). Beyond 4 μm, the aircraft’s own thermal radiation gradually increases in proportion [47], with radiation attenuation primarily attributed to atmospheric absorption effects. Atmospheric H_2_O and CO_2_ exhibit strong absorption bands near wavenumbers of approximately 1590 cm^−1^ and 2349 cm^−1^, respectively. Energy in the red spectral region is absorbed at these points. Concurrently, the atmosphere absorbs to varying degrees across other wavenumber ranges, resulting in overall reductions in peak intensities. Multiple atmospheric gases possess distinct absorption bands; their combined effects introduce greater fluctuations in the red spectrum, altering the shape of the entire spectral line.

It should be noted that the vertical axis in Figure 8 employs brightness temperature (K), while Figure 12 and Figure 15 present results in terms of radiative intensity [W/(sr·cm^−1^)]. Brightness temperature *T_B_* is defined as the temperature of an equivalent blackbody that would emit the same spectral radiance as the observed target. It is related to radiative intensity $I_{\eta }$ through Planck’s law $\;T_{B}=hc\eta /[k_{B}\cdot ln(\frac{2hc^{2}\eta^{3}}{I_{\eta }}+1)]$. This conversion enables meaningful comparison between the experimental measurements (brightness temperature) and the numerical simulation results (radiative intensity) presented in this study.

Based on the simulated data (Figure 9), the U.S. standard atmosphere serves as a reference atmosphere. Its transmittance curve reflects typical mid-latitude atmospheric composition, exhibiting low transmittance in strong absorption bands (CO_2_ around 2349 cm^−1^, H_2_O around 1590 cm^−1^ and 3756 cm^−1^, O_3_ around 1041 cm^−1^), while exhibiting high transmittance in atmospheric windows (833–1250 cm^−1^, 2380–2857 cm^−1^, 4000–5000 cm^−1^). Compared to winter, northwest China exhibits higher water vapor content during summer, resulting in lower transmission in water vapor absorption bands and overall transmission slightly lower than in winter, particularly in the longwave infrared region. Coastal China shows stronger absorption than northwest China across all water vapor absorption bands, leading to lower overall transmission.

Major atmospheric absorbing gases (especially water vapor) significantly absorb radiative transmission. Regional and seasonal variations primarily stem from changes in water vapor content, with coastal areas exhibiting strongest absorption in summer and northwest regions weakest in winter. Furthermore, selective atmospheric absorption does not create new spectral features but strongly modulates the original trail radiation spectral features. Accurate spectral feature extraction requires atmospheric correction models to subtract these effects. These conclusions provide theoretical support for designing future field experiment protocols.

## 5. Conclusions

This study investigates the flow characteristics and infrared radiation spectrum of an aero-engine wake flow field through numerical simulation. It analyzes the influence mechanisms of three key factors—fuel–air ratio, exhaust temperature, and atmospheric absorption effects—on the wake field’s flow properties and infrared radiation spectrum. The primary conclusions are as follows:

(1) The fuel–air ratio significantly affects the temperature distribution, Mach number evolution, and molar fractions of characteristic gases (CO_2_ and H_2_O) in the wake field. Under high fuel–air ratios, the core region exhibits higher temperatures, a more concentrated Mach number distribution, and a smaller expansion angle. The molar fractions of CO_2_ and H_2_O increase with rising fuel–air ratio, directly leading to enhanced peak intensities and broadened half-widths in the infrared spectrum. Within the fuel-lean operating regime (f = 0.025–0.031, f/f_st_ ≤ 0.45), the fuel-to-air ratio is one of the key factors governing the spectral radiation characteristics of the wake.
(2) The exhaust temperature exerts a “global amplification” effect on the temperature distribution within the wake field. Under high-temperature conditions, the core region of the wake exhibits higher temperatures and slower decay, with spectral radiation intensity increasing exponentially with temperature. Temperature exerts a negligible influence on CO_2_ and H_2_O molar fractions, indicating that component distribution is primarily governed by combustion chemical equilibrium and diffusion dilution rather than temperature regulation. Temperature elevation significantly enhances radiation transition probabilities in the characteristic bands of H_2_O and CO_2_, inducing spectral redshift and broadening effects.
(3) Atmospheric effects exhibit strong band-selective absorption of tail flow infrared radiation, particularly near absorption bands such as CO_2_ (2349 cm^−1^), H_2_O (1590 cm^−1^, 3756 cm^−1^), and O_3_ (1041 cm^−1^), where transmittance is notably low. Regional and seasonal atmospheric conditions (e.g., water vapor content) significantly influence radiation transport processes, with strongest absorption occurring in coastal summer regions and weakest in northwest winter regions. Atmospheric absorption does not generate new spectral features but strongly modulates the original radiation spectrum, necessitating restoration through atmospheric correction models.

This study did not consider the potential impact of intermediate reaction pathways on fuel-rich combustion conditions. Future work will incorporate multi-step reaction mechanisms and validate them through field experiments. Additionally, the influence patterns of parameters such as bypass ratio, combustion efficiency, engine count, presence of afterburners, and stealth measures on the infrared spectral features of engine exhaust plumes will be key areas for subsequent research. Furthermore, this study analyzed the three factors individually and did not consider the synergistic or antagonistic effects of multi-factor combined operating conditions (e.g., high fuel–air ratio combined with high exhaust temperature) on the wake infrared spectral features; multi-factor coupling effects will be investigated in future work through combined-condition simulations and field experiments.

## Figures and Tables

**Table 1:** Calculation method for nozzle component concentration.

Ingredients	Mole (mol)	Mole Fraction (%)
N_2_	$0.79n_{a}$	$\frac{0.79}{1+0.82f}$
O_2_	$0.21n_{a}-17.75n_{f}$	$\frac{0.21-3.07f}{1+0.82f}$
CO_2_	$12n_{f}$	$\frac{2.08f}{1+0.82f}$
H_2_O	$11.5n_{f}$	$\frac{1.99f}{1+0.82f}$

**Table 2:** Molar Fraction of BEM-2 engine nozzle components and flow field parameters.

Flow Field Parameters			Gas Component Fraction						
Pressure, Pa	Temperature, K	Mach	O_2_	CO_2_	CO	N_2_	H_2_	OH	HCl
2.88 × 10^5^	1960	2.35	0.4	0.136	0.115	0.096	0.056	0.056	0.192

**Table 3:** Distribution of integral intensity errors within the three spectral bands.

Spectrum Band	2.70–2.95 μm	1.20–1.45 μm	1.50–5.50 μm
Test (W/sr)	25.8	83.5	235.0
Calculate (W/sr)	33.2	77.0	238.4
Error (%)	28.7	7.78	1.45

## Data Availability

The data presented in this study are available upon request from the corresponding author.
